# Microbial drivers of root plasticity

**DOI:** 10.1111/nph.70371

**Published:** 2025-07-21

**Authors:** Francisco Dini‐Andreote, Darren M. Wells, Jonathan A. Atkinson, Brian S. Atkinson, Omri M. Finkel, Gabriel Castrillo

**Affiliations:** ^1^ Department of Plant Science, Huck Institutes of the Life Sciences The Pennsylvania State University University Park PA 16802 USA; ^2^ The One Health Microbiome Center, Huck Institutes of the Life Sciences The Pennsylvania State University University Park PA 16802 USA; ^3^ School of Biosciences University of Nottingham Sutton Bonington LE12 5RD UK; ^4^ Department of Plant & Environmental Sciences, The Alexander Silberman Institute of Life Science The Hebrew University of Jerusalem Edmond J. Safra Campus – Givat Ram Jerusalem 9190401 Israel

**Keywords:** microbiota controlling root development, plant nutrition, plant–microbiota interactions, root plasticity in soil, soil heterogenicity

## Abstract

Soils are highly heterogeneous and dynamic systems, experiencing a constant flow of plant root exudates and moisture fluctuations that affect nutrient distribution, soil physicochemical properties, and microbial metabolisms. Plant roots adjust their development within the soil matrix (growth, branching, root angle, and anatomical features) by integrating local environmental conditions with physiologically informed signals. These physiological responses and the adaptability of roots are commonly defined as plasticity. Although genetically determined, root plasticity is modulated by local fluctuations in water and nutrient availability, environmental changes, and soil properties as well as by the root‐associated microbiota. Roots and their vicinity are colonized by taxonomically and functionally diverse microbial species. Specific members within these communities can establish chemical communication with plants via root‐derived signals, thereby tapping into the plant's hormonal and developmental network, influencing root plasticity. Given that most root traits associated with plasticity have been discovered under axenic conditions, our knowledge of the multiple potential mechanisms by which members of the root‐associated microbiota modulate root plastic responses is still limited. In this review, we explore the recent progress in this field and identify specific avenues for future research toward advancing molecular and ecological understanding of microbial‐mediated root plasticity in terrestrial systems.

## Resumen

Los suelos son sistemas altamente heterogéneos y dinámicos, que experimentan un flujo constante de exudados radiculares y fluctuaciones de humedad que afectan la distribución de nutrientes, las propiedades fisicoquímicas del suelo y el metabolismo microbiano. Cuando crecen en el suelo, las raíces de las plantas ajustan su desarrollo (por ejemplo, su crecimiento, ramificación, el ángulo con que emergen las raíces, sus características anatómicas) integrando las condiciones ambientales locales del suelo con señales fisiológicas en las raíces. Estas respuestas de adaptabilidad de las raíces a las condiciones cambiantes del suelo se definen comúnmente como plasticidad de las raíces. Aunque está genéticamente condicionada, la plasticidad radicular se modulada también por las fluctuaciones locales en la disponibilidad de agua y nutrientes en el suelo, los cambios ambientales y en las propiedades del suelo, así como por la microbiota asociada a las raíces. Las raíces y sus alrededores están colonizados por especies microbianas taxonómica y funcionalmente diversas. Miembros específicos de estas comunidades pueden establecer comunicación química con las plantas a través de señales derivadas de las raíces, aprovechando así la red hormonal y de desarrollo de la planta, lo que influye en la plasticidad radicular. Dado que la mayoría de los factores radiculares asociados con la plasticidad se han descubierto en condiciones de esterilidad, sin la presencia de microorganismos, nuestro conocimiento de los múltiples mecanismos potenciales mediante los cuales los miembros de la microbiota radicular modulan las plasticidad de las raíces es aún limitado. En esta revisión, exploramos los avances recientes en este campo e identificamos líneas específicas para futuras investigaciones que podrían promover la comprensión molecular y ecológica de la plasticidad radicular mediada por microbios en sistemas terrestres.

## Abstract (he)

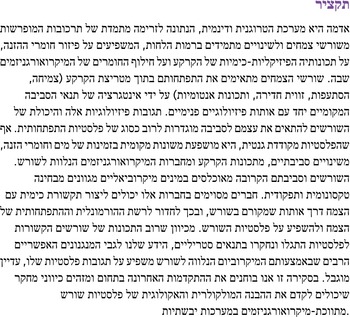

## Resumo

Solos são sistemas altamente heterogêneos e dinâmicos, experimentando um fluxo constante de exsudatos de raízes de plantas e flutuações de umidade que afetam a distribuição de nutrientes, as propriedades físico‐químicas do solo e o metabolismo microbiano. Raízes de plantas ajustam seu desenvolvimento dentro da matriz do solo (crescimento, ramificação, ângulo da raiz, características anatômicas) integrando condições ambientais locais com sinais fisiologicamente informados. Essas respostas fisiológicas e a adaptabilidade das raízes são comumente definidas como plasticidade. Embora geneticamente determinada, a plasticidade radicular é modulada por flutuações locais na disponibilidade de água e nutrientes, mudanças ambientais e propriedades do solo, bem como pela microbiota associada à raiz. Raízes e suas proximidades são colonizadas por espécies microbianas taxonomicamente e funcionalmente diversas. Membros específicos dentro dessas comunidades podem estabelecer comunicação química com plantas via sinais derivados da raiz, assim acessando a rede hormonal e de desenvolvimento da planta, influenciando a plasticidade radicular. Dado que a maioria das características radiculares associadas à plasticidade foi descoberta sob condições axênicas, nosso conhecimento dos múltiplos mecanismos potenciais pelos quais membros da microbiota associada à raiz modulam as respostas plásticas radiculares ainda é limitado. Nesta revisão, exploramos o progresso recente neste tema e identificamos áreas específicas para futuras pesquisas visando promover o avanço da compreensão molecular e ecológica da plasticidade radicular mediada por microrganismos em sistemas terrestres.

**Table:** 

	Contents	
	Summary	52
I.	Introduction	53
II.	Plant–microbe interactions: modulation of the rhizosphere as microbial habitats	53
III.	Biotic effects of the rhizosphere microbiota on root system architecture	54
IV.	Microbial effects on root responses to environmental signals	57
V.	Contribution of the microbiota to endogenous root responses to soil nutrient availability	59
VI.	Integration of biotic signals at the level of root system anatomy	60
VII.	Concluding remarks and future perspectives	61
	Acknowledgements	62
	References	62

## I. Introduction

The high spatial and temporal heterogeneity of soil properties has conditioned the evolution of plant roots, which have adapted to navigate and extract resources from soil matrices that are highly heterogeneous and dynamic in terms of physical and chemical gradients (García‐Palacios *et al*., 2012). This multiscale and spatiotemporal amalgamation of soil characteristics emerges from the inherent complexity of the soil matrix, which encompasses physicochemical and biological properties, weather fluctuations, and anthropogenic effects, such as soil management practices and pollution (Piotrowska‐Długosz *et al*., 2019; Nunan *et al*., 2020; Nyengere *et al*., 2023; Sünnemann *et al*., 2023; Zhang *et al*., 2023; Mooney *et al*., 2024).

Soils are dynamic systems continuously experiencing variations in physicochemical and biological properties mediated by the constant influx of root exudates and organic matter transformation, combined with soil moisture fluctuations on daily and seasonal scales. These dynamics impact the soil's water availability, pore space and oxygen tension, and micro‐ and macro‐aggregate structure. The spatial distribution of soil aggregates and physicochemical gradients results in the development of heterogeneous microenvironments within the soil matrix (Gravuer *et al*., 2020; Jarvis *et al*., 2024; Shabtai *et al*., 2024). These spatiotemporal gradients in soil structure and properties drive fluctuations in microbial metabolisms, leading to shifts in the biosynthesis of active exometabolites and the transformation and solubilization of essential nutrients (Sasse *et al*., 2018). Faced with these continual fluctuations in resource availability and the physical and biological constraints within the soil matrix, terrestrial plants have evolved diverse root morphological characteristics (i.e. architecture and anatomical traits) and the ability to adjust their growth, branching patterns, angle, and cellular composition to maximize resource uptake and effectively respond to environmental gradients (Morris *et al*., 2017).

The ability of roots to sense and respond to soil heterogeneity is genetically determined by endogenous root developmental programs, which are largely modulated by the integration of root responses to local changes in water and nutrient availability, soil density, pH, temperature, O_2_ and CO_2_ levels, and the soil biota (Yetgin, 2024). This malleable root physiological adaptability is commonly referred to as root plasticity – namely, the ability of roots to adjust their growth (e.g. root length, branching, depth) in response to local soil conditions/signals, including microbially derived metabolites (Colombi *et al*., 2024). However, from a strictly classical perspective, the term ‘root plasticity’ also encompasses what can be described as root elasticity. To accurately describe the actual responses of roots to fluctuations in soil resources and conditions, it has been proposed that ‘plasticity’ should apply only to root phenotypes that are retained even after the initial stimulus is attenuated (e.g. as the root reaches another area of the soil), whereas ‘elasticity’ refers to root phenotypes that are immediately reversed when the stimulus is no longer present (Colombi *et al*., 2024). For example, roots can exhibit plasticity if plants continue to develop lateral branches in response to high‐phosphate nutrient patches, whereas elasticity occurs when roots reduce the development of lateral roots upon sensing available sources of phosphate (Colombi *et al*., 2024).

Most studies related to the discovery of root traits associated with plasticity and elasticity have been carried out under axenic conditions and in response to chronic exposure to isolated abiotic factors (e.g. nutrient deficiency, water stress). As a result, these studies often overlook the natural cycles of multiple stresses that roots encounter on their trajectories through the soil as well as potential mechanisms of root responses mediated by the soil‐ and root‐associated microbiota. Ecological interactions between plants and microbes have existed since the beginning of the evolution of land plants (Remy *et al*., 1994) and are thought to have facilitated early root development and the evolution of the plant immune system (Martin *et al*., 2017; Han, 2019; Lee & Hwang, 2021). Despite the majority of the root microbiota being acquired horizontally from the soil, some microbial taxa are consistently found to be associated with roots across diverse plant species, soil systems, and environmental conditions (Lemanceau *et al*., 2017), suggesting common modes of microbial recruitment and providing evolutionary evidence for plant–microbe symbiosis.

Given the intricate associations of plant roots with microbes, the root–microbiota system can be integrated and conceptually considered an ecological unit (Berg *et al*., 2024). Within this framework, it is intrinsically assumed that the modulation of plant physiology – including the coordination of endogenous root developmental programs regulating plasticity and elasticity – is at least, in part, modulated by microbial metabolisms. While in recent years, some studies have shown the role of the root microbiota in modulating root architecture (Finkel *et al*., 2020; Gonin *et al*., 2023; Gifford *et al*., 2024), the molecular processes underlying how specific microbial taxa influence root physiology in response to cycles of resource availability or multiple distinct stresses remain poorly explored.

## II. Plant–microbe interactions: modulation of the rhizosphere as microbial habitats

The term ‘rhizosphere’ coined by Hiltner (1904) refers to the narrow zone of soil directly influenced by plant roots (Hiltner, 1904). This zone encompasses both the vertical stratification and horizontal heterogeneity of the soil matrix, resulting in variable gradients of physicochemical properties that dynamically determine the formation of microbial microhabitats (i.e. variations in soil microaggregates, pH, O_2_ tension, chemical gradients, and availability of distinct organic substrates). Chemical gradients in the rhizosphere are modulated by the active influx of plant‐derived substrates and the active metabolism of microbes engaged in plant–microbe interactions (Cordovez *et al*., 2019).

A considerable proportion of the total amount of carbon (C) fixed by the plant (i.e. photosynthates – *c*. 20–40% based on total C) is released by plant roots as exudates, mucilage (i.e. polysaccharides secreted by the root cap), cell wall materials (e.g. cellulose, hemicellulose, pectin, lignin), and lysates (derived from the breakdown of root cells and tissues) (Canarini *et al*., 2019). Root exudation encompasses a wide range of primary (e.g. sugars, organic acids, amino acids, vitamins, nucleotides and fatty acids) and specialized metabolites (e.g. flavonoids, alkaloids, phenolics, terpenoids) (Stassen *et al*., 2021; Wang *et al*., 2022; McLaughlin *et al*., 2023). These alterations in soil properties – combined with the effect of root physical growth and respiration – determine the ‘rhizosphere effect’ (Lv *et al*., 2023), which is reflected in distinct compositions of bacterial and fungal communities compared to those in the bulk soil (Lundberg *et al*., 2012; Edwards *et al*., 2015; Fitzpatrick *et al*., 2018; Thiergart *et al*., 2020). The mechanisms by which root exudation modulates the soil microbiota, and how this influences plant health, are major focal areas of current research efforts (e.g. Huang *et al*., 2019; Pantigoso *et al*., 2025). Variations in the chemical composition and flux of root exudates occur across plant species (Dhungana *et al*., 2023; McLaughlin *et al*., 2023), developmental stages (Santangeli *et al*., 2024), nutritional status (Haase *et al*., 2007; Carvalhais *et al*., 2011), and even across distinct root orders and structures within the same plant (Aufrecht *et al*., 2022; Loo *et al*., 2024).

Most studies on the rhizosphere microbiota have treated the rhizosphere as a homogenous unit (i.e. due to standardized rhizosphere and root exudate sampling protocols; Barillot *et al*., 2013). However, to understand the molecular and chemical mechanisms underlying plant–microbe interactions, it is essential to consider the distinct microhabitats defined by gradients of resource availability in the rhizosphere (Box 1). For instance, this can be achieved by using sampling efforts based on preestablished root classification systems according to metrics such as root diameter (coarse or fine roots), branching (primary, secondary, tertiary), and function (i.e. absorptive and transportive). Alternatively, plant mutants lacking morphological features like root branches or root hairs could be examined. Classification by branching order and by functional traits often allocates fine roots into either absorptive fine roots (root orders 1 and 2) or transportive fine roots (root orders 4 and above) (Fitter, 1982; Pregitzer *et al*., 2002; McCormack *et al*., 2015). Importantly for the microscale modulation of resources in the plant rhizosphere, absorptive fine roots are the most metabolically active, dynamically structuring physicochemical gradients within the rhizosphere (Reinhold‐Hurek *et al*., 2015). Corroborating this, studies have shown that absorptive fine roots support greater bacterial abundance and mycorrhizal fungal colonization (Guo *et al*., 2008b; King *et al*., 2021), and exert greater selection on the rhizosphere microbiota than higher‐order roots (i.e. transportive fine roots) (King *et al*., 2021). These effects are mostly caused by detectable differences between fine root functional groups related to root respiration rates, root hair densities, morphology, absorptive capacities, and life span, all of which affect carbon substrate availability in these microhabitats (Guo *et al*., 2008a,b; Valenzuela‐Estrada *et al*., 2008; Makita *et al*., 2009; Gu *et al*., 2011; McCormack *et al*., 2015).

Box 1 **Defining ecological niches in the plant–rhizosphere**

Conceptually, the term ‘ecological niche’ applies to individual species (micro‐ and macroorganisms) and it is defined as an *n*‐dimensional hypervolume where the boundaries or ‘dimensions’ are set by biotic and abiotic variables that exert an influence on the species' survival, reproduction, and persistence (*sensu* Hutchinson, 1957, 1978). Applied to rhizosphere ecology, this concept suggests that the dynamic coexistence of multiple species in a complex system requires variable physicochemical gradients that differentially influence microbial fitness and selection. By contrast, homogeneity in microhabitat structures would favor competitive exclusion, thus reducing species diversity. Moreover, the concept of ecological niche is a valuable autecological construct (Holt, 2009), which provides an essential conceptual tool for understanding species' range limits and boundaries of species competition and coexistence (Soberón, 2007). Distinct species in the rhizosphere are differentially affected by the dynamic temporal and spatial heterogeneity of chemical gradients determined by plant metabolism and the consumption and modification of resources performed by multiple species across the rhizosphere microhabitats. These interactive, resource‐related variables align with Hutchinson's concept of ‘bionomics’ (Hutchinson, 1978), directly linking microbial metabolism with the uptake and transformation of root‐derived carbon substrates. In turn, this complex metabolic and microbial landscape modulates root development and growth, as roots integrate multiple modulatory signals from their immediate environment.

## III. Biotic effects of the rhizosphere microbiota on root system architecture

**Fig. 1:**
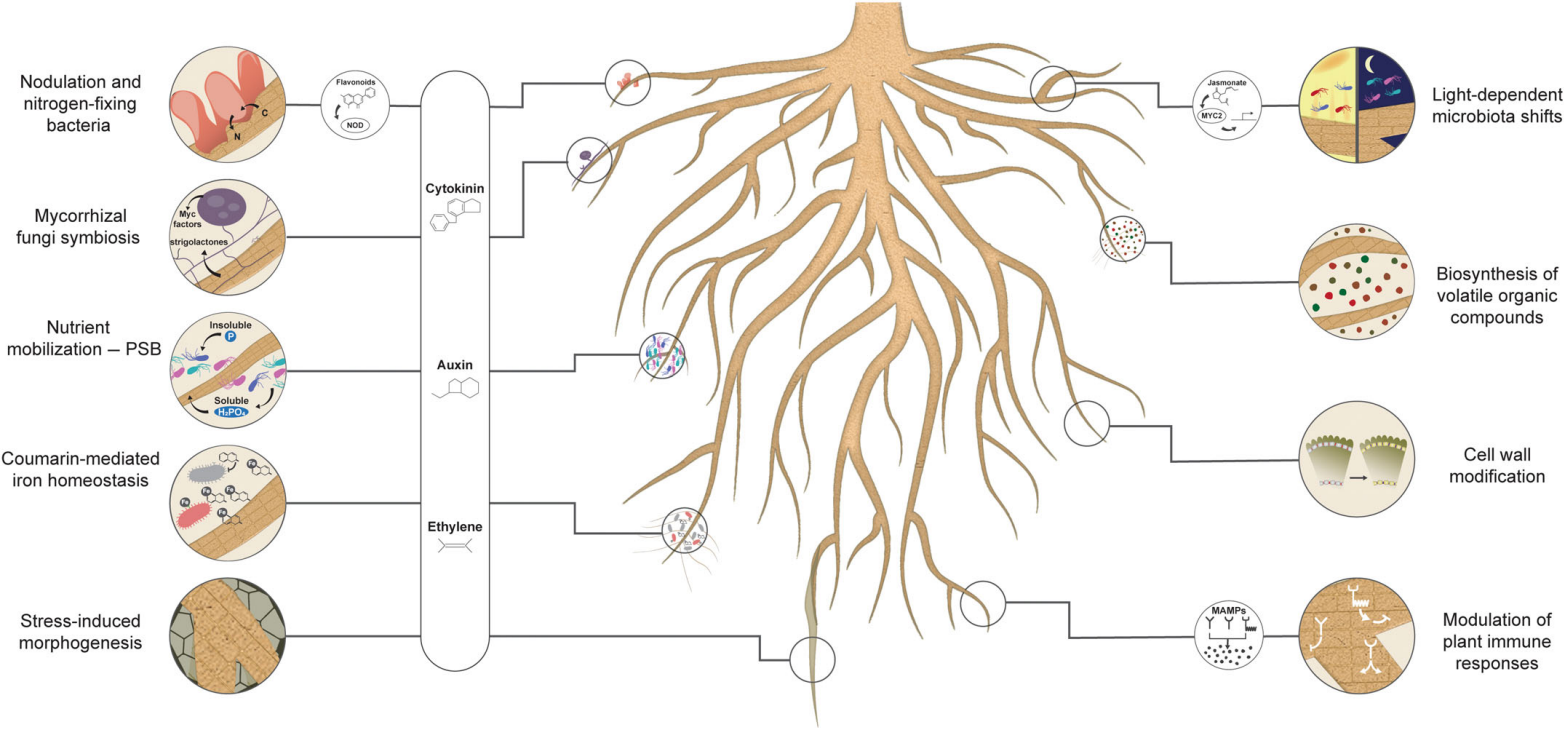
Schematic representation of plant–microbe interactions and microbial metabolism that influence root development and nutrient uptake. The figure displays the main identified mechanisms by which microbes interfere with the plant's endogenous developmental and nutrient uptake programs, resulting in changes in root development and plasticity, and plant nutritional status. MAMPs, microbe‐associated molecular patterns; NOD, Nod factors; PSB, phosphate‐solubilizing bacteria.

For root plasticity to be adaptive (i.e. increase plant fitness), the plant needs to integrate myriad environmental signals that vary in time and space within the rhizosphere and respond in a coordinated fashion, amending its developmental program both locally and systemically. This challenge becomes even more complex when the signals are biotic (Fig. 1; Table 1). While plants must incorporate the surrounding microbiota into their developmental responses, similar to how they respond to chemical cues (Atkinson & Urwin, 2012), the microbiota themselves face adaptive pressures, as both the abiotic environment and the development of the root affect microbial fitness. This creates a delicate ecological discourse, where numerous members within the microbiota – some beneficial, others harmful – manipulate root development to establish a more hospitable microhabitat. Simultaneously, the root attempts to perceive reliable abiotic cues as mechanisms to combat harmful microbes and, in some cases, foster beneficial ones (Trivedi *et al*., 2020).

**Table 1:** Examples of mechanisms of microbiota‐mediated changes in root plasticity.

Mechanism	Microbial groups	Effects on root plasticity	References
Auxin/cytokinin modulation	*Pseudomonas*, *Bacillus*, *Xanthomonadales*, *Variovorax* mycorrhizal fungi, complex synthetic communities	Alters auxin/cytokinin balance affecting lateral root formation, root elongation	Finkel *et al*. (2020); Tzipilevich *et al*. (2021)
Ethylene modulation	ACC deaminase‐producing bacteria (e.g. *Variovorax*), endogenous ethylene inducing bacteria	Alters root hair and lateral root density	Glick (2014); Pandey *et al*. (2021); Gonin *et al*. (2023)
Immune evasion and suppression	*Xanthomonadales* (e.g. *Dyella japonica*), protease‐secreting bacteria	Dampens immune responses, promotes root growth by degrading MAMPs (e.g. flg22)	Ma *et al*. (2021); Teixeira *et al*. (2021); Eastman *et al*. (2024); Ordon *et al*. (2025)
Nutrient mobilization	Phosphate‐solubilizing bacteria (PSB, e.g. *Flavobacteria*), AM fungi, *Colletotrichum tofieldiae*	Enhances lateral root branching in nutrient‐depleted zones	Hiruma *et al*. (2016); Castrillo *et al*. (2017); Lidbury *et al*. (2020); Liu *et al*. (2024)
Volatile organic compounds (VOCs)	*Bacillus* spp., Trichoderma	Modulates primary root growth and root hair density	Zhang *et al*. (2007)
Cell wall modification	*Pseudomonas simiae* WCS417, pathogens (e.g. *Verticillium*), complex synthetic communities	Alters suberin lamellae, Casparian strip formation, and cortical aerenchyma	Fröschel *et al*. (2021); Salas‐González *et al*. (2021); Verbon *et al*. (2023)
Symbiotic nodulation	*Rhizobia*, *Frankia*	Induces lateral root and nodule formation via Nod factors and flavonoid signaling	Bozsoki *et al*. (2020); Libourel *et al*. (2023); Tao *et al*. (2024)
Stress‐induced morphogenesis	Drought‐adapted microbiota (e.g. Enterobacter)	Promotes deeper rooting via ethylene/KMBA signaling	Zélicourt *et al*. (2018)
Coumarin‐mediated iron homeostasis	Microbiota shifts by coumarins	Influences root hair elongation and iron scavenging via shifts in the microbiota	Stringlis *et al*. (2018); Harbort *et al*. (2020); Paffrath *et al*. (2024)
Light‐dependent microbiota shifts	Shoot‐light‐responsive commensals	Modulates root branching via MYC2/jasmonate signaling under low PAR	Hou *et al*. (2021)

ACC, 1‐aminocyclopropane‐1‐carboxylate; AM, arbuscular mycorrhiza; flg22, flagellin peptide flg22; KMBA, 2‐keto‐4‐methylthiobutyric acid; MAMP, microbe‐associated molecular patterns; PAR, photosynthetic active radiation; PSB, phosphate solubilizing bacteria.

At the molecular level, the capability of microorganisms to manipulate root development depends on the existence of receptors in root cells that bind microbe‐derived molecules. This is the basis for the plant's general non‐self‐response systems (Keppler *et al*., 2024), among which are the pattern recognition receptors (PRRs) that can trigger immune responses (Shu *et al*., 2023). Ligands for these PRRs, known as microbe‐associated molecular patterns (MAMPs), can induce severe root growth arrest, which is used as a marker for the activation of effective defense responses (Ma *et al*., 2021). While this can be seen as a part of a general growth‐defense trade‐off (He *et al*., 2022), this phenotype – usually observed in confined, homogenous settings (e.g. agar plates) – may also represent an avoidance mechanism. That is, in a larger, heterogeneous root system, resources can be allocated away from root meristems affected by a pathogen, toward uninfected parts of the root system.

Immune responses and their related developmental phenotypes can also be silenced or dampened by specific members of the rhizosphere microbiota. Such immune‐suppressive microbes have been identified by measuring their impact on root length (Ma *et al*., 2021). This effect often appears to be mediated by bacterial secretion of proteases, which help the bacteria recycle secreted proteins and peptides, with the added benefit of cloaking them from the plant's immune system. Such evasion of immune recognition, in both plants and mammals, was shown to be mediated by the bacterial alkaline protease *AprA*, which effectively degrades flagellin monomers (flg22), preventing the activation of FLS2 receptors in plants (and TLR5 receptors in mammals) (Bardoel *et al*., 2012; Pel *et al*., 2014). This dampening of flg22‐derived immunity is widespread in rhizosphere bacteria (Ma *et al*., 2021; Teixeira *et al*., 2021), particularly within the order Xanthomonadales, including both commensal and phytopathogenic *Xanthomonas* strains (i.e. *Xanthomonas campestris* pv *vesicatoria* (Xcv 85‐10), *X. campestris* pv *campestris* (Xcc 8004), and *Xanthomonas hortorum* pv *pelargonii* (Xhp)) (Ordon *et al*., 2025). The genes *dssAB*, components of an ABC transporter, which are highly conserved in Xanthomonadales, are required for immunosuppression. In addition, some Xanthomonadales species can also secrete peptidases that eliminate immunogenic peptides. For example, the endophytic root colonizer *Dyella japonica* evades immune recognition by cleaving flg22 with a type II secreted subtilase (Eastman *et al*., 2024; Ordon *et al*., 2025). This activity can also function as a ‘common good’, dampening immune responses for the benefit of other rhizosphere members (Ma *et al*., 2021; Teixeira *et al*., 2021), although it was still shown to confer a competitive advantage to the strains carrying these metabolic genes (Ordon *et al*., 2025).

Another major class of microbially produced molecules that roots can perceive and respond to are phytohormones. Several soil microbes are able to synthesize phytohormones (e.g. auxins, cytokinins, ethylene) (Arshad & Frankenberger, 1991; Dodd *et al*., 2010; Tzipilevich *et al*., 2021; Mekureyaw *et al*., 2022). Phytohormones may have originated as interspecies communication molecules in bacteria and algae, preceding their role in plant physiology (Van Overbeek, 1940; Segev *et al*., 2016). Microbial phytohormone production and its effect on root development have been documented since the 1930s, first observed in the related systems of nodule‐forming rhizobia (Thimann, 1936) and crown gall‐forming *Agrobacterium* (Link & Wilcox, 1937), and a few years later, in free‐living bacteria (Spaepen & Vanderleyden, 2011; Zamioudis *et al*., 2013). Since different root architectural features influence microbial colonization (Galindo‐Castañeda *et al*., 2024; Wang *et al*., 2024), the ability to reshape roots into a more hospitable microhabitat is likely adaptive for microbes. Microbially produced auxin, for instance, acts in a similar way to exogenously applied auxin. It causes the arrest of primary root growth and stimulates lateral root formation and root hair growth (Banda *et al*., 2019). These changes may benefit bacteria by making plant‐derived nutrients more accessible (Spaepen *et al*., 2014; Mashiguchi *et al*., 2019). Bacterial auxin also interacts negatively with the salicylic acid signaling pathway in plants, thus inhibiting immune responses (Kunkel & Harper, 2018). For example, the gram‐positive *Bacillus velesensis*, a model biocontrol agent, produces auxin in response to immune‐derived reactive oxygen species. The presence of auxin promotes bacterial colonization, likely due to induced root developmental changes. In this way, bacterial colonizers can use plant immune responses to promote colonization rather than diminish it (Tzipilevich *et al*., 2021). In addition, auxins can also be utilized by plant pathogens to enhance virulence. For example, *Ralstonia solanacearum*, which proliferates in xylem vessels causing bacterial wilt, depends on auxin to promote infection. This phytopathogen inhibits primary root growth and induces root hair formation, in addition to altering early xylem differentiation in a manner dependent on the HrpG effector, to promote xylem colonization. This is evidenced by auxin‐insensitive plant mutants showing resistance to this pathogen (Zhang *et al*., 2024). Auxin response manipulation is also a common strategy among viral pathogens (Müllender *et al*., 2021). Therefore, bacterial‐derived auxin cannot be seen as beneficial or detrimental in and of itself, as both mutualistic and pathogenic microorganisms utilize it to favor plant tissue colonization.

Bacteria in the rhizosphere not only produce but also degrade plant hormones, which can act as carbon and nitrogen sources for microbial metabolisms. For example, genomic information associated with auxin production and auxin degradation is widespread across bacterial genomes (Conway *et al*., 2022). So far, this has been identified to be mediated by distinct operons: the *iaa* operon, which encodes the anaerobic conversion of IAA to 2‐aminobenzoyl‐CoA (Thoenen *et al*., 2023), and the *iac* operon (Leveau & Gerards, 2008) or the *iad* operon (Finkel *et al*., 2020; Conway *et al*., 2022; Ma *et al*., 2023), which aerobically degrade IAA. Nearly, all strains/isolates within the ubiquitous root colonizer species *Variovorax paradoxus* carry an *iad* operon, and their presence in the rhizosphere can result in dampening effects of auxin‐producing bacteria (Finkel *et al*., 2020; Conway *et al*., 2022). Microbes in the rhizosphere can also interact with plant auxin regulation via alternative pathways. For example, bacterial volatile organic compounds (VOCs) can induce auxin production by the plant, triggering cell expansion (Zhang *et al*., 2007).

The bacterial gene *acdS*, which encodes 1‐aminocyclopropane‐1‐carbxylate (ACC)‐deaminase, can also interfere with plant hormone signaling (Glick, 2014). Since ACC is the precursor of ethylene, rhizosphere bacteria with the *acdS* gene can reduce plant ethylene‐mediated stress responses by reducing ethylene levels (Glick, 2014). Since stress response typically involves growth arrest (Bechtold & Field, 2018), ACC deaminase is viewed as a plant growth‐promoting factor (Chen *et al*., 2013; Glick, 2014; Saikia *et al*., 2018; Herpell *et al*., 2023). However, unlike auxin degradation, which appears to primarily target auxins produced by bacteria, ACC‐deaminase acts on endogenous ACC, depriving the plant of perceiving it as a stress signal. This can stimulate growth in the short term, particularly under abiotic stress (Saikia *et al*., 2018), although it may undermine long‐term plant fitness. This ‘ethylene paradox’ is further complicated by observations that some bacteria enhance plant stress tolerance via ethylene biosynthesis. For instance, an *Enterobacter* species isolated from desert plants was shown to induce stress tolerance in Arabidopsis and alfalfa by synthesizing 2‐keto‐4‐methylthiobutyric acid (KMBA), which is converted into ethylene (Zélicourt *et al*., 2018). Hence, different studies have documented bacterial protection against abiotic stress through both increasing and decreasing ethylene levels. Many bacteria also influence root branching through mechanisms linked to the plant's response to ethylene in a manner partially independent of auxin signaling since these effects persist in mutant plants with deficiencies in auxin signaling and perception (Gonin *et al*., 2023).

## IV. Microbial effects on root responses to environmental signals

### 1. Gravity and light

After germination and radicle emergence, root architecture starts to be modulated by two abiotic environmental stimuli: gravity and light. The ability of roots to sense gravity is facilitated by specialized starch‐filled plastids, called statoliths, located at the bottom of the columella cells in the root cap (Nishimura *et al*., 2023). These statoliths shift their position in response to the root's orientation, resulting in an asymmetric lateral gradient of the phytohormone auxin between the top and bottom of the root (Chauvet *et al*., 2019). This gradient leads to root curvature, aligning the root's growth with the gravity vector (Zhang *et al*., 2019). This gravity‐sensing system allows the plant to penetrate the soil matrix and direct root development. Gravity also influences the angles of emergence and growth of different root types, such that different root types exhibit different angles, which reduces self‐competition and increases the volume of soil explored (Kirschner *et al*., 2024). The dependence of gravity sensing on local auxin perception suggests microorganisms producing or degrading auxin at specific locations along the root axis can affect gravitropism. Considering the significance of this mechanism for root development, it is surprising that the influence of the resident root microbiota on it has not yet been mechanistically documented. Only a handful of experiments have been performed analyzing the assembly of seed and root microbiota under simulated microgravity conditions. These experiments have indicated that, under reduced gravity, the bacterial community composition of seeds and roots changes in wheat plants (Cui *et al*., 2022, 2023).

In addition to gravity, light also orients root growth and development away from the soil surface, following a negative phototropism pattern (Izzo *et al*., 2022). The root response to light is regulated by the tight coordination between the cryptochrome, the phytochrome, and phototropin classes of light receptors, along with the polar localization of auxin transporters (Matsuoka & Tokutomi, 2005; Halliday *et al*., 2009; Legris *et al*., 2019; Palayam *et al*., 2021). Interestingly, roots also integrate light input signals from the shoots through the activation of phytochrome B (Lee *et al*., 2016). In *Arabidopsis thaliana*, regulatory pathways controlling responses to light and root bacterial commensals were shown to be connected between roots and shoots. When shoots detect low photosynthetically active radiation (PAR), mechanisms controlling root bacterial community composition are activated. In turn, shoot growth impairment caused by low PAR is mitigated by root microbial commensals through microbiota‐ and light‐dependent growth‐defense trade‐off mechanisms. This involves the transcription factor MYC2, an important regulator of the jasmonate signaling pathway (Hou *et al*., 2021). Thus, the root microbiota contributes to the root's integration of light‐quality signals from the shoot to prioritize either plant defense or growth, which could improve plant survival and performance (Hou *et al*., 2021).

### 2. Water availability

Water is heterogeneously distributed in the soil due to vertical stratification, physicochemical properties, and variations in aggregates and pore size. As a result, the soil matrix typically consists of a mix of pores – some filled with water, others covered by a thin water film, and others that are air‐filled pores. Plant roots adapt to this uneven water distribution through both plastic and elastic responses (Kirkham, 2014). Roots modify their growth trajectory toward regions of higher water availability through hydrotropism (Dietrich, 2018), a process mediated by the abscisic acid (ABA) pathway and by Ca^2+^ signals activated asymmetrically in specific tissues within the root elongation zone. These signals lead to enhanced cell expansion on the side of the root exposed to low water potential, bending the root toward the water source (Shkolnik *et al*., 2018; Miyazawa & Takahashi, 2019). Soil water distribution also conditions the radial pattern of lateral root initiation. When primary roots grow through the soil matrix and are exposed to uneven water contents, lateral roots initiate preferentially on the side of the root with higher water potential as part of an adaptive response called hydropatterning (Bao *et al*., 2014). This response involves the activation of auxin biosynthesis and transport on the side of the root in contact with water (Bao *et al*., 2014). Hydropatterning is also regulated by posttranslational covalent attachment of small ubiquitin‐like modifier (SUMO) proteins to the lateral root regulator *AUXIN RESPONSE FACTOR 7* and ethylene levels on the side of the root exposed to air, leading to an inhibition of lateral root formation on this side (Orosa‐Puente *et al*., 2018; Scharwies *et al*., 2025).

**Fig. 2:**
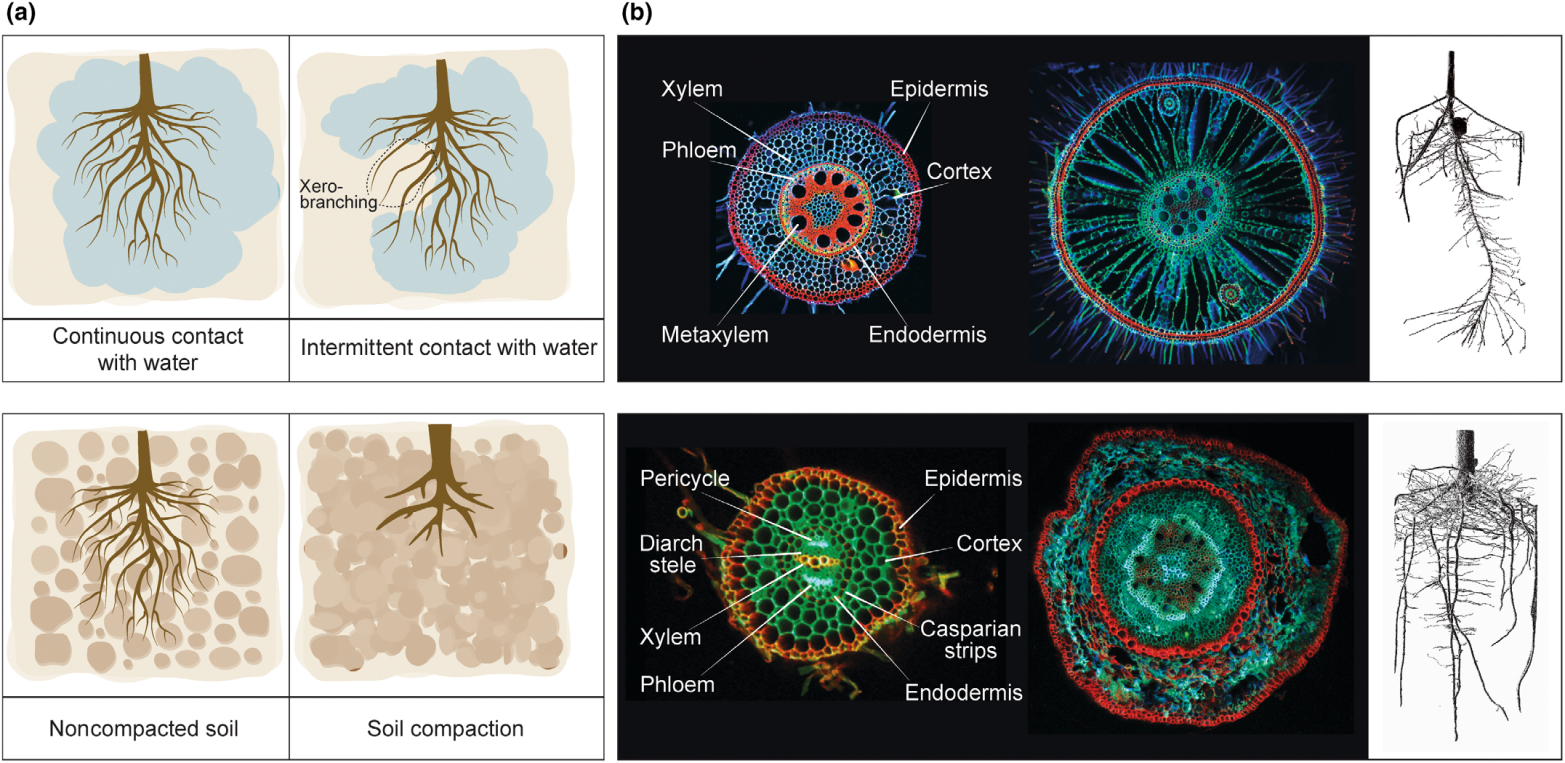
Overview of plant‐root phenotypic (architecture and anatomical) plasticity in soil. (a) Schematic representations of root plasticity in response to intermittent contact with water, leading to xerobranching (upper panel) and soil compaction (lower panel). (b) Confocal microscopy images displaying root cross‐sections highlighting differences in the anatomical arrangement between monocotyledonous and dicotyledonous species. The upper images (left to right) illustrate root anatomical cross‐sections of *Zea mays* (maize) and *Oryza sativa* (rice) (monocotyledonous species), and the lower images (left to right) display root anatomical cross‐sections of *Solanum lycopersicum* (tomato) and *Vitis vinifera* (grapevine) (dicotyledonous species). In both cases, the blunt‐ended arrows identify and name the different tissue layers and relevant anatomical features in the root. Side images in each inset panel are X‐ray computed tomography (CT) scan images of *Z. mays* (maize) (monocotyledonous, upper image) and *V. vinifera* (grapevine) (dicotyledonous, lower image). X‐ray CT enables the visualization of 3D structures based on differences in X‐ray attenuation (see Mairhofer *et al*., 2017; Piovesan *et al*., 2021).

Another microscale root response to water availability occurs when root tips grow into dry, air‐filled soil pores. Under these conditions, when root tips temporarily lose contact with water, branching is suppressed by a response called xerobranching (Orman‐Ligeza *et al*., 2018; Fig. 2a). This inhibitory response is distinct from hydropatterning and is controlled by ABA (Mehra *et al*., 2022). High levels of ABA during this temporary phase of low water availability induce closure of plasmodesmata in the outer root tissues, leading to reduced radial movement of auxin from the epidermis to the lateral root founder cells in the pericycle, resulting in suppression of lateral root formation (Mehra *et al*., 2022). If water scarcity is more severe, roots grow downward, causing lateral roots to shift from shallow to steeper in exploiting the soil profile. This change in the root growth pattern is caused by enhanced gravitropism in response to water stress and is called xerotropism (Wexler *et al*., 2024). This response is different from hydrotropism and is, to a certain extent, controlled by auxin (Wexler *et al*., 2024). Although all these processes take place in close contact with the rhizosphere microbiota, which is known to change its composition in roots in response to water availability and can influence auxin and ABA metabolism (Finkel *et al*., 2020; Conway *et al*., 2022; Ryabova *et al*., 2024), the contribution of the microbiota to root responses to microscale water heterogeneity remains unexplored. However, several studies have associated changes in root architecture – for example, increased number and length of lateral roots; increased total root volume, area, diameter, and widening of xylem vessels – with the presence of certain microbial taxa described in close association with roots during these responses to water availability (Ali *et al*., 2022; Qi *et al*., 2022; Gu *et al*., 2024; Yue *et al*., 2024).

### 3. Soil compaction

During the penetration through the soil matrix, roots encounter changes in the soil's physical properties, such as an increase in bulk density, which generally increases with soil depth due to gravitational pressure (Panagos *et al*., 2024). This vertical increase in soil density is associated with a reduction of pore connectivity (DeJong‐Hughes *et al*., 2001), resulting in a significant increase in the soil's resistance to penetration and a reduction in gas and water conductivity (Berisso *et al*., 2012). In response, plants reduce the number and length of lateral roots and the total root length, thus progressively exploring a smaller volume of soil with detrimental effects on nutrient and water uptake (Giuliani *et al*., 2024; Fig. 2a). The root response to high soil density is generally conserved in monocots and eudicots and is based on the reduced diffusion of gases in compacted soils. Specifically, ethylene – a gaseous phytohormone released at the root tips – accumulates within and near the roots in compacted soils due to low diffusion (Pandey *et al*., 2021). As a result, this microscale accumulation of ethylene induces changes in the synthesis and transport of auxin and ABA, leading to the inhibition of root elongation and formation of fewer but longer root hairs, and radial thickening of roots (Pandey *et al*., 2021; Huang *et al*., 2022; Pandey & Bennett, 2024). In rice, increased ethylene concentration in compacted soils was shown to trigger the expression of *WUSCHEL‐RELATED HOMEOBOX 11*, which activates the emergence and growth of crown roots to compensate for the inhibition of roots in deeper soil layers (Li *et al*., 2024). In specific cases, such as maize and wheat, to improve the mechanical resistance of the root tissue to allow penetration of high‐density soils, the high concentration of ethylene induces the formation of a specialized lignified tissue called multiseriate cortical sclerenchyma in monocotyledons and the lignification of other root tissues (Schneider *et al*., 2021). These modifications in anatomical and metabolic traits are expected to affect the composition and functions of root‐associated microbial communities. For example, roots of maize plants grown in high‐density soils were shown to be less colonized by the beneficial endophytic basidiomycete fungus *Piriformospora indica* (Hosseini *et al*., 2018). Moreover, increases in soil bulk density are correlated with a decrease in the number and size of nodules in soybean plants (Siczek & Lipiec, 2011). This effect may be driven by atypical ethylene accumulation around the roots growing through compacted soil, as rhizobia‐mediated nodulation (Nod) is inhibited by the external application of ethylene due to changes in root sensitivity to *Nod* factors (Oldroyd *et al*., 2001). Furthermore, atypical ethylene accumulation in and around roots can also dynamically impact nonrhizobial root–bacteria interactions. For example, a high concentration of ethylene artificially supplied to roots was shown to increase bacterial alpha diversity in the rhizosphere (Chen *et al*., 2020). Since microbes were shown to influence ethylene levels both positively and negatively (Gonin *et al*., 2023), the rhizosphere microbiota probably influences the capability of roots to penetrate dense soil. However, aside from some fragmented information, relatively little is known about how the root development program integrates microbial function during the root's physiological response to high soil density.

## V. Contribution of the microbiota to endogenous root responses to soil nutrient availability

Plant root development is closely tied to nutrient availability in the soil. Plants optimize nutrient uptake through morphological adaptations, including enhanced lateral root branching and root hair elongation. These adaptations increase the soil area explored by the roots, maximizing access to nutrients (Aibara & Miwa, 2014; Lynch *et al*., 2023) and the interface for biotic interactions. Microorganisms can modulate root branching (as described above), shaping the rhizosphere thereby improving nutrient accessibility for the plants (Gonin *et al*., 2023). Reciprocally, changes to root branching patterns dynamically influence the composition and function of the root microbiota, with lower‐order absorptive roots exerting stronger selection and supporting more bacterial cells per root biomass than higher‐order ones (King *et al*., 2021).

The soil nutrient status also modulates shifts in the chemical composition of root exudates, shaping the microbiota assembly and functioning in the rhizosphere. For instance, in a study where a switchgrass prairie was amended with either N, P or both nutrients (Baker *et al*., 2024), N amendment strongly altered exudation patterns, favoring N‐containing metabolites, such as amino acids, at the expense of organic acids and aromatic compounds, which were enriched in the nutrient‐poor soil. These changes corresponded to targeted alterations in the rhizosphere microbiota. Interestingly, the P amendment in this study had a very moderate effect on both exudation and microbiota composition, although several studies report low P soils inducing root secretion of organic acids, strigolactones, and phosphatases (e.g. Wang *et al*., 2021).

Disentangling the microbiota's direct influence on plant abiotic stress tolerance is challenging because the stressor itself simultaneously impacts both the plant host and its microbial communities. Therefore, this endeavor requires conceptual understanding of, and the ability to manipulate, the plant's endogenous stress response mechanisms. For example, the PHOSPHATE STARVATION RESPONSE (PSR) system, regulated by the v‐myb avian myeloblastosis viral oncogene homolog (MYB) transcription factors PHOSPHATE STARVATION RESPONSE 1 (PHR1), orchestrates plant physiological adaptation to P‐limitation (Bustos *et al*., 2010; Madison *et al*., 2023), whereas the transcriptional response to nitrogen limitation involves NIN‐like proteins and nitrate transporters (NRTs) (Liu *et al*., 2022; Wang *et al*., 2023). The PSR system was recently shown to regulate specific plant–microbe interactions. Activation of the PSR system directly influences the composition and function of root‐associated microbiota (Castrillo *et al*., 2017; Herrera Paredes *et al*., 2018; Finkel *et al*., 2019). Additionally, the PHR1–RALF–FERONIA axis suppresses plant immunity under P limitation. This can facilitate colonization by beneficial microbes that enhance P uptake (Castrillo *et al*., 2017; Tang *et al*., 2022) but can also allow opportunistic pathogens to infect the plant (Finkel *et al*., 2019). Since immunity is suppressed under P limitation, some fungal pathogens have evolved mechanisms to induce PSR in plants even under phosphate‐replete conditions. For instance, NUDIX proteins in *Magnaporthe* and *Colletotrichum* fungi hydrolyze inositol pyrophosphate to induce PSR and promote susceptibility to infection (McCombe *et al*., 2025). PSR transcription factors like PHR1 are also essential for maintaining arbuscular mycorrhizal (AM) symbiosis by regulating genes involved in fungal colonization and phosphate uptake (Das *et al*., 2022). The symbiosis of ancient plant progenitors with arbuscular mycorrhizal fungi (AMF) was established *c*. 470 million years ago (Ma) (Remy *et al*., 1994) and is directly associated with the evolution of vascular plants. Notably, an estimated 70–90% of land plants are able to form symbiotic associations with mycorrhizas (Martin & van der Heijden, 2024). AM extend hyphal networks to scavenge P beyond the root depletion zone (Smith *et al*., 2011), absorbing P through Pi transporters and shuttling it as polyphosphate toward the root, where it is hydrolyzed by fungal endopolyphosphatases (Zhao *et al*., 2024). Nonmycorrhizal plants rely on alternative plant–fungal partnerships. For instance, *Helotiales* fungi have been shown to mimic AM traits in *Arabis alpina*, facilitating P acquisition through colonization of the root endosphere (Almario *et al*., 2017). These partnerships also tend to rely on PSR. In *A. thaliana*, the endophytic fungus *Colletotrichum tofieldiae*, a close relative of the pathogenic fungus *Colletotrichum incanum*, was shown to deliver P to the shoot in a mode of action that also depends on the PSR system (Hiruma *et al*., 2016).

Nearly all P in soils is immobilized within organic or inorganic complexes (Lidbury *et al*., 2020). Bacteria and fungi, in particular AM, play an important role in P acquisition by solubilizing phosphate and by mobilizing organic P, whereas other microbial groups compete with the plant under P‐limited conditions (Zoysa *et al*., 1997; Finkel *et al*., 2019). Phosphate‐solubilizing bacteria, which belong to diverse taxa, secrete organic acids and phosphatases that release phosphorus from mineral complexes (Lidbury *et al*., 2020). Based on metagenomic data, it was shown that *c*. 50% of soil bacteria contain known genes associated with P‐scavenging enzymes, which are often found in higher abundance in high‐pH soils (Lidbury *et al*., 2017). The phylum Bacteroidetes, in particular the genus *Flavobacteria*, have been implicated as key players in P mobilization in and around plant roots (Johansen & Binnerup, 2002), exhibiting phosphatase activity under a wide range of P conditions (Lidbury *et al*., 2020). In addition, a unique and complex phosphate acquisition regulon was recently described in a *Flavobacterium* isolated from *Brassica napus*, which was shown to be generally enriched in plant‐associated Bacteroidetes. Another recent study in *B. napus* showed that an endophytic *Flavobacterium*, enriched under P limitation, solubilizes phosphate and alleviates plant phosphate limitation (Liu *et al*., 2024).

In addition to P, the microbiota play a pivotal role in providing nitrogen (N) to plants. Dinitrogen fixation by Rhizobium in leguminous plants is one of the most prominent examples of a microbial symbiont altering host development. Nitrogen‐fixing symbioses evolved *c*. 100 Ma in a common ancestor of the four angiosperm orders Fabales, Fagales, Cucurbitales, and Rosales (the Nitrogen‐Fixing Nodulation clade) (Libourel *et al*., 2023). In Fabales, particularly within the legume family (Fabaceae), where plants typically associate with alpha‐ and betaproteobacteria, the symbiosis was retained in most species. However, among Fagales, Cucurbitales, and Rosales, plants form symbiotic relationships with Actinomycetota of the genus Frankia (Griesmann *et al*., 2018), though symbiosis was lost in many species (Libourel *et al*., 2023). This symbiosis has now been extensively studied for over a century (Nobbe *et al*., 1891), and it serves as the paradigm for microbial nutritional symbionts taking over the host's developmental program: nodule formation, the development of specialized organs that host bacteroids (Poole *et al*., 2018).

The chemical cues and molecular mechanisms underlying nodule symbioses are now well understood, although aspects such as symbiont specificity (Yu *et al*., 2024; Haskett *et al*., 2025) and enforcement against cheating (Westhoek *et al*., 2021) are still not entirely elucidated. Specificity is enforced by both host and symbiont factors. Host factors are often flavonoids, which are perceived by *Lys*R family *Nod*D proteins in the rhizobial partner, inducing the expression of strain‐specific Nod genes. These genes encode enzymes involved in the synthesis of Nod factors, which are lipochitooligosaccharides that bind LysM receptor complexes in the plant (Bozsoki *et al*., 2020), thus promoting Nod. The effects of Nod factors are not restricted to the specific symbiont. In fact, their activity alters plant exudation, with consequences for the recruitment and functioning of the rhizosphere microbiota (Zgadzaj *et al*., 2016; Tao *et al*., 2024). Thus, nitrogen‐fixing symbiosis is not only a pairwise plant–microbe interaction, but also involves other players. Protists, for example, were recently shown to facilitate the movement of nitrogen fixing bacteria toward roots (Hawxhurst *et al*., 2023; Micciulla *et al*., 2024). Moreover, microbially mediated shifts in host development in response to nitrogen deficiency are not restricted to legumes and nitrogen fixers. In maize plants, for example, specific flavonoids were found to enrich for Oxalobacteriaceae in the rhizosphere, thus ameliorating plant nitrogen stress (Yu *et al*., 2021).

In addition to flavonoids, several other plant‐secreted heterocyclic molecules play a role in maintaining nutritional symbioses with microorganisms. For example, coumarins, a diverse class of molecules, appear to play a dual critical role under iron deprivation. On one hand, coumarins act as iron chelators involved in iron mobilization and shuttling (Paffrath *et al*., 2024). On the other hand, coumarins also affect the plant microbiota (Voges *et al*., 2019), via antimicrobial effects (Stringlis *et al*., 2018) and by influencing iron homeostasis in microbes in the rhizosphere (Harbort *et al*., 2020; McRose *et al*., 2023). The multiple roles of these compounds (flavonoids, phytoalexins, and coumarins) – acting directly in plant stress alleviation and indirectly via the modulation of root‐associated microbiota – are currently an active area of study. This includes particular focus on their spatial distribution across root tissue and their relation to root development.

## VI. Integration of biotic signals at the level of root system anatomy

Root developmental plasticity is also coordinated by transcriptional responses at the cellular level that contribute to phenotypic variations in root anatomy (Fig. 2b). These responses are highly cell‐type‐specific, with each root cell type exhibiting distinct molecular signatures that shape their responses to environmental biotic and abiotic signals (Iyer‐Pascuzzi *et al*., 2011; Long, 2011). Specifically, biotic signals include MAMPs (i.e. exogenous molecules produced by bacteria, fungi, oomycetes, nematodes, and parasitic plants) and DAMPs (i.e. endogenous compounds released from plant tissues upon wounding that occurs during infection) (Manosalva *et al*., 2015; Hegenauer *et al*., 2016; Yu *et al*., 2017). Both MAMPs and DAMPs are perceived by plant PRRs localized to the plasma membrane, triggering a signaling cascade that can be associated with root cellular reprogramming and adaptive changes in root architecture (Kawa & Brady, 2022). In brief, these changes can include variations in cortical cell size and cortical cell layers, which determine the thickness of the root diameter (influencing nutrient absorption and anchorage), cortex size (affecting the storage and nutrient transport), the formation of root cortical aerenchyma (influencing oxygen transport and diffusion, plant–biotic associations, Galindo‐Castañeda *et al*., 2019), and the development of apoplastic barriers in the endodermis (i.e. Casparian strip, suberin lamellae) (associated with the movement of water and nutrients and protection against pathogen infection) (Thomas *et al*., 2007; Andersen *et al*., 2015; Singh *et al*., 2021; Fig. 2b).

One of the most studied examples of microbially induced variations in root anatomy is the symbiotic relationship between plants and AMF. It has been shown that the perception of AMF colonization in outer root tissues triggers yet unknown intraradical signaling mechanisms that induce cell cycle reactivation in cortical cells (reviewed in detail in Russo & Genre, 2021). This process results in the induction of complete mitosis and/or endocycle in inner cortical cells, which occurs before AMF colonization and arbuscule formation in cortical cells (see Carotenuto *et al*., 2019a,b; Russo *et al*., 2019a,b). Conversely, the mechanism by which legume plants engage in symbiotic associations with nitrogen‐fixing bacteria is also associated with the modulation of root anatomical traits. In brief, Nod is not only dependent on Nod factors secreted by nitrogen‐fixing bacteria but also involves several other factors (e.g. plant hormonal regulation, calcium signaling). This results in signal transduction leading to the expression of multiple genes, which produce coding and noncoding regulatory RNAs that modify plant developmental programs. Such modifications are associated with cell wall remodeling and the microbially mediated induction of lateral root and nodule formation (Roux *et al*., 2014; Traubenik *et al*., 2020).

The microbially induced variations in root anatomy can be directly associated with the metabolism of individual members of the plant microbiota or with the overall structure of the plant‐associated microbiota. For example, recent studies showed that the bacterium *Pseudomonas simiae* WCS417 can affect different root cell types of *A. thaliana* by altering cell wall biogenesis and suberin deposition inside the root (Verbon *et al*., 2023). Moreover, a recent study elucidated a regulatory mechanism of endodermal differentiation mediated by the root microbiota with consequences for plant nutrient homeostasis. In brief, the root microbiota influences the deposition of endodermal diffusion barriers, which control the plant mineral nutrient homeostasis. This microbially induced effect is mediated by the plant's ABA signals (Salas‐González *et al*., 2021). Interestingly, the development of these apoplastic barriers in root endodermal tissues forms a protective physical barrier that hinders pathogen invasion (Thomas *et al*., 2007; Andersen *et al*., 2015; Singh *et al*., 2021). However, studies have shown that infection by specific phytopathogens, such as *Verticillium longisporum*, can downregulate genes associated with suberin biosynthesis and Casparian strip formation in the endodermis. This weakens the diffusion barrier, allowing the fungus to enter the vasculature (Fröschel *et al*., 2021). Furthermore, *V. longisporum* can also induce transdifferentiation – a process in which nonxylem cells are reprogrammed into xylem cells – resulting in significant changes to the cell transcriptome and cellular architecture (Reusche *et al*., 2012). Last, a recent study showed that specific species within the sorghum‐root microbiota can induce changes in root cellular anatomy – specifically, root endodermal suberization and aerenchyma formation. The authors reported these microbially mediated plastic changes in root anatomy to be partially responsible for the suppression of root infection by the parasitic weed *Striga hermonthica* (Kawa *et al*., 2024).

## VII. Concluding remarks and future perspectives

The plant's capacity to sense and respond to environmental signals through the soil matrix – including abiotic and biotic cues – is directly associated with root plastic and elastic responses aimed at optimizing plant fitness and survival (Figs 1, 2). This intricate process involves a complex interplay of plant genetic mechanisms, physiological responses, and ecological factors associated with the active recruitment and metabolism of microbial communities in the rhizosphere. While the importance of microbial influence on root plasticity is increasingly recognized, a significant knowledge gap persists regarding the specific mechanisms by which microbes mediate these responses. Future research must prioritize dissecting the molecular pathways through which microbial metabolites, signaling molecules, and direct interactions impact root development. Emerging technologies such as single‐cell RNA sequencing and spatial transcriptomics now enable unprecedented resolution in mapping plant–microbe interactions, allowing researchers to identify cell‐type‐specific responses to microbial metabolites. When combined with advanced imaging techniques like fluorescence *in situ* hybridization coupled with confocal microscopy and X‐ray CT, these approaches can reveal how microbial colonization patterns correlate with root architectural modifications at microhabitat scales.

Understanding how specific microbial taxa modulate root hormone signaling, nutrient uptake, and stress responses is an active area of research, although relatively less attention has been given to integrating these with measures of root phenotypic plasticity – for instance, variation in root architecture and anatomical traits. Spatial metabolomics approaches, including MALDI‐TOF imaging and desorption electrospray ionization mass spectrometry (DESI‐MS), can provide crucial insights by mapping the distribution of microbially derived metabolites across root tissues. These techniques will be particularly valuable for identifying key metabolic signals that mediate root–microbe interactions, such as phytohormone‐like molecules or VOCs that influence root growth trajectories, branching patterns, and tissue differentiation. Furthermore, the integration of functional genomics with high‐resolution phenotyping platforms offers exciting opportunities for both fundamental discovery and applied outcomes. From a breeding perspective, understanding the genetic basis of root–microbe interactions could inform selection strategies for crops that better recruit beneficial microbiota or respond more effectively to microbial signals. Similarly, to advance our understanding of how root plasticity and elasticity interact with the microbiota at the scale relevant for a microbial cell, future research should adopt finer resolution approaches, examining microbiota functions associated with distinct root orders and microhabitats. For instance, the integration of functional genomics and transcriptomics approaches can elucidate the plant's molecular responses to microbial signals, revealing the intricate signaling networks that govern root plasticity, especially in the understudied responses, such as gravity, microscale water availability, and soil compaction. Understanding the interplay between microbial communities, root anatomical features, and environmental conditions at the microscale will require advanced methodologies, including single‐cell transcriptomics, spatial metabolomics, and *in situ* microbiome imaging techniques.

Despite significant progress, our understanding of root plasticity and elasticity has predominantly emerged from controlled studies focusing on isolated abiotic stresses, and controlled, homogenous growth media. These systems overlook the complex reality plant roots experience in natural soils, where multiple stresses frequently interact and fluctuate. Future research should increasingly employ experimental systems that simulate realistic, dynamic soil environments to unravel root responses under conditions closer to natural ecosystems or agricultural settings. Ultimately, advancing fundamental knowledge of microbially mediated mechanisms modulating root plasticity will contribute toward the development of applied strategies that harness the beneficial effects of the soil microbiome to enhance crop productivity and resilience, bridging the gap between fundamental research and practical applications in optimizing plant performance in the context of global environmental changes.

## Competing interests

None declared.

## Author contributions

FD‐A, OMF and GC contributed to the planning and writing of the manuscript, with the input of the other authors. FD‐A, OMF and GC designed the figures, which use CT scan and confocal microscopy images generated by DMW, JAA and BSA.

## Disclaimer

The New Phytologist Foundation remains neutral with regard to jurisdictional claims in maps and in any institutional affiliations.
